# Identifying Genomic Alterations in Small Cell Lung Cancer Using the Liquid Biopsy of Bronchial Washing Fluid

**DOI:** 10.3389/fonc.2021.647216

**Published:** 2021-04-26

**Authors:** Jinfang Zhai, Songyan Han, Qinxiang Guo, Binbin Shan, Jing Wang, Yanrong Guo, Guoping Tong, Chang Zhao, Yuan Li, Qiao Han, Xiaoqin An, Ruiqing Yue, Li Wang, Tingting Guo, Zhentian Liu, Yaping Xu, Jianqiang Li, Weihua Yang

**Affiliations:** ^1^Department of Respiratory and Critical Care Medicine, The Second Hospital of Shanxi Medical University, Taiyuan, China; ^2^Department of Respiratory Ward One, Shanxi Provincial Cancer Hospital, Taiyuan, China; ^3^Geneplus-Beijing, Beijing, China

**Keywords:** small cell lung cancer, liquid biopsy, bronchial washing fluid, TMB, cfDNA

## Abstract

**Objective:** With the rapid development of cancer genomics and immunomics, some new treatments of small cell lung cancer (SCLC) are emerging. However, there are limitations to the clinical use of tumor tissue. Our study aimed to evaluate the potential use of bronchial washing fluid (BWF) in the liquid biopsy of SCLC.

**Methods:** Twenty-one extensive SCLC (ES-SCLC) patients were enrolled in this study. For all patients, four sample types, BWF supernatant (BWFs), BWF precipitate (BWFp), plasma and tumor tissue, were collected before receiving chemotherapy, and one type, plasma, was collected after chemotherapy. All samples were conducted to NGS using the 1021-gene panel. The concordance rates of genomic profiling using NGS in the four types of samples were evaluated. Multiple clinical information was analyzed for correlation.

**Results:** We successfully tested 20 BWFs samples, 21 BWFp samples, 21 tumor tissue samples, 20 pre-treatment plasma, and 13 post-treatment plasma of these 21 patients. The detectability of somatic mutations was 100% for BWFs, BWFp, tumor tissues, and post-treatment plasma, and only one pre-treatment plasma was absent with any mutation. Matched tumor tissue, BWFs, BWFp, and pre-treatment plasma samples were subsistent for 19 patients. For these patients, 204 genomic alterations were identified in tissue samples, while 189 (92.6%), 175 (85.5%), and 163 (79.9%) alterations were detected in the matched BWFs, BWFp, and pre-treatment plasma, respectively. Moreover, we found that the three tumor markers associated with SCLC have a lower sensitivity than genomic alterations. The endocrine resistance pathway was found enriched in hyponatremia patients which may be related to the hyponatremia. The TMBs of BWF, BWFp, and pre-treatment plasma samples all had a strong correlation with that of tissue samples. Both the VAF and the MVAF of mutations in post-treatment plasma were less than those in pre-treatment plasma, which was in accordance with the evaluation of curative effect.

**Conclusions:** For ES-SCLC patients, the liquid biopsy of BWF showed a highly potential advantage to identify DNA alterations, which suggested that genomic analysis of BWF liquid biopsy may have clinical value as a supplement for tissue and blood detection. Through the restricted validation, it can be widely used in routine clinical practice.

## Introduction

Small-cell lung cancer (SCLC), ~3–15% of all lung cancers, is an exceptionally lethal subtype of lung cancer due to its high invasiveness and metastatic proneness (1). Majority of SCLC patients have missed their surgical treatment opportunity when first consulting in outpatient clinics. Chemotherapy and radiotherapy have been the main treatments of SCLC for decades, but the 5-year survival was not improved due to lack of therapeutic innovation (2). In recent years, immune checkpoint blockage (ICB)-based clinical trials have brought new insight into the management of ES-SCLC and the therapy exceeded the median overall survival (OS) by 1 year for the first time (3, 4). However, the therapeutic efficacy is heterogeneous and related markers are urgently needed. Apart from the well-known PD-1/PD-L1 expression, several emerging markers based on genomic analysis such as tumor mutational burden (TMB) and microsatellite instability (MSI) (5, 6) have a promising performance in predicting ICB efficacy for multiple tumor types. For advanced patients with inaccessible tissue biopsy, peripheral blood-based liquid biopsy provides an alternative way to assess the ICB curative effect genomically (7).

Liquid biopsy has been used extensively in multiple malignancies to analyze and monitor cancer in various body effluents including blood, pleural and ascitic fluid, urine, and cerebrospinal fluid instead of tumor tissue (8, 9). It has the potential to identify actionable alterations non-invasively and can overcome both spatial and temporal tumor heterogeneity not addressed by tissue biopsy and can follow subclonal evolution through multiple blood draws (10). The analysis of circulating cell-free DNA (cfDNA) in plasma is showing promise in several other important emerging cancer applications, including detection of postsurgical minimal residual disease for prediction of recurrence, real-time monitoring of response to treatment, and early detection of cancer (8). In addition, several studies (11–13) have highlighted the usage of circulating tumor DNA (ctDNA) in SCLC and suggest that ctDNA sequencing can delineate genomic landscape, subclonal architecture, and genomic evolution of SCLC. Meanwhile, bronchial washing (BW) is a minimally invasive procedure developed on the basis of fiberoptic bronchoscopy. The BW procedure yields fluids (BWF) (8, 10) that may contain tumor-derived DNA fragments. Considering the direct adjacency to bronchi, BWF might also be a desirable semantide for the analysis of tumor genomics.

In order to provide more implication for the clinical application of liquid biopsy, we enrolled 21 ES-SCLC patients in this study and collected tumor tissue, peripheral blood, and BWF from each patient prior to chemotherapy. High-throughput sequencing was performed to evaluate the genomic status of each sample. A high consistency about the mutational spectrum was found, while ICB-related markers and TMB status exhibited a significant correlation in different specimens. Despite the similarity, we also found that BWFs might be a superior media of liquid biopsy on the basis of ctDNA abundance and detectability of tumor-derived mutations. Collectively, this study for the first time assessed the utility of BWFs in the genomic analysis of ES-SCLC, and the findings may give significative notions to future diagnosis and therapeutic decision for ES-SCLC.

## Materials and Methods

### Clinical Cohort

In this study, 21 ES-SCLC patients were enrolled in 2019. Clinicopathological data, demography, tumor histopathological results, and biochemical tumor markers associated with SCLC were collected from each patient. This study was approved by the institutional review board of Shanxi Provincial Cancer Hospital. All subjects provided informed, written consent before study-related procedures. This study was conducted in accordance with the Declaration of Helsinki.

### Sample Processing and DNA Extraction

Tumor tissue, BWF, and peripheral blood were collected from all patients before chemotherapy, and peripheral blood was collected after EP regimen chemotherapy. Supernatant and precipitate were separated from BWF, while blood cell and plasma were separated from peripheral blood. For each patient, all pre-treatment samples were collected within 3 days, post-treatment samples within 2 weeks after two cycles of EP regimen (cisplatin combined with Etoposide) chemotherapy. In those 21 cases, 18 cases had visible lesions under bronchoscopy. The lesions were directly washed with 20 ml 0.9% saline, and the irrigation fluid was recovered by suction. These 18 tissue samples were gathered by direct forceps biopsy. For 3 patients that had no visible lesion under bronchoscopy, chest enhanced CT was performed, then the bronchi near the lesion were flushed for recovering the flushing fluid, and TBNA (transbronchial needle aspiration) was performed to the station 4R lymph node with shorter diameter longer than 1.5 cm to gather tissue samples. The recovery fluid had a minimal volume of 6 ml. We centrifuged the fluid at 600 g for 10 min to separate the supernatant and the precipitant. More than 10 ml of peripheral blood (PB) was collected from each patient using cell-free DNA blood collection tubes (Streck, Omaha, NE, USA) at room temperature before receiving chemotherapy and 2 weeks after the end of two cycles of chemotherapy. PB samples were centrifuged at 2,500 g for 10 min, then moved to microcentrifuge tubes and centrifuged at 16,000 g for 10 min to remove remaining cellular debris. Peripheral blood lymphocytes (PBLs) generated from the first centrifugation were gathered as germline control samples. Pre-plasma, post-plasma, PBLs, BWFs, and BWF precipitate (BWFp) sample were stored at −80°C. The tissue DNA, BWFp DNA, and PBL DNA were extracted using the DNeasy Blood & Tissue Kit (Qiagen, Hilden, Germany). Circulating cfDNA was isolated from 0.6 to 1.8 mL plasma and 1.5 to 3 mL BWFs using QIAamp Circulating Nucleic Acid Kit (Qiagen). The DNA concentration was measured using the Qubit fluorometer (Invitrogen, Carlsbad, CA, USA) dsDNA HS kit, and the sizes of cfDNA fragments were assessed using the Agilent 2100 BioAnalyzer and DNA HS kit (Agilent Technologies, Santa Clara, CA, USA). One pre-treatment plasma (pre-plasma), one post-treatment plasma (post-plasma), and one BWF supernatant (BWFs) were unqualified for low DNA amount.

### Library Preparation and Hybridization Capture-Based Sequencing

Before library preparation, 1 μg of BWFp DNA, tissue DNA, and PBL DNA was sheared into 200–250-bp fragments with a Covaris S2 instrument (Woburn, MA, USA). Indexed next-generation sequencing (NGS) libraries were constructed from PBL DNA, BWFp DNA, tissue DNA, BWFs DNA, and plasma DNA (cfDNA) with KAPA Library Preparation Kit (Kapa Biosystems, Wilmington, MA, USA). For cfDNA, after end repairing and A-tailing, well-designed adapters with unique identifiers (UIDs) were ligated to both ends of the double-stranded cfDNA fragments. The SeqCap EZ Library system (Roche NimbleGen, Madison, WI, USA) was used for target enrichment. All libraries were hybridized to custom-designed biotinylated oligonucleotide probes (IDT, Coralville, IA, USA) covering 1.6 Mbp of the genome. The captured genomic regions included 1,021 genes (shown in Supplementary Table 1) from multiple sources. We selected common oncogenic driver genes (14), genes in the key signaling pathway, drug-therapy relevant genes, and high-frequency mutant regions recorded in the Catalog of Somatic Mutations in Cancer (COSMIC, http://cancer.sanger.ac.uk/cosmic) and The Cancer Genome Atlas (TCGA, https://cancergenome.nih.gov/). Captured DNA fragments were amplified after hybrid selection and then pooled into several multiplexed libraries. Sequencing was performed using the MGIseq-2000 sequencing system (BGI, Shenzhen, China) following the manufacturer's instruction.

### Raw Data Processing

After removal of terminal adaptor sequences and low-quality reads (>50% N rate, >50% bases with Q <5), remaining reads were mapped to the reference human genome (hg19) and aligned using Burrows–Wheel Aligner (version 0.7.12-r1039, http://bio-bwa.sourceforge.net/) with default parameters, followed by duplicate read identification using the Picard MarkDuplicates tool (https://software.broadinstitute.org/gatk/documentation/tooldocs/4.0.3.0/picard_sam_ markduplicates_MarkDuplicates.php). Base quality recalibration and local realignment were conducted by the Gene Analysis Toolkit (GATK, https://www.broadinstitute.org/gatk/). The median effective depths of tissue, BWFs, BWFp, pre-plasma, and post-plasma were 897 × (*n* = 20, range 440–1,485), 4,276.5 × (*n* = 21, range 1,825–5,907), 4,189 × (*n* = 20, range 1,902–6,172), and 893 × (*n* = 21, range 557–2,624).

### Mutation Identification, Functional Analysis, and Tumor Mutational Burden

Somatic single-nucleotide variants (SNVs) and insertions or deletions of small fragments (Indels) were called by MuTect algorithm (https://software.broadinstitute.org/gatk/documentation/tooldocs/3.8-0/org_broadinstitute_gatk_tools_walkers_cancer_m2_MuTect2.php). PBL sequencing data were used to filter germline mutations. All reliable alterations were supported by ≥5 high-quality sequencing reads (mapQthres > 30, baseQthres > 30). Multiple single-nucleotide polymorphism databases (dbsnp, https://www.ncbi.nlm.nih.gov/projects/SNP/; 1000G, https://www.1000genomes.org/; ESP6500, https://evs.gs.washington.edu/; ExAC, http://exac.broadinstitute.org/; self-built SNP database) were used to ensure the accuracy of somatic detection. An online tool WebGestalt (http://www.webgestalt.org) was used for gene-enrichment analysis (15). TMB was calculated based on 1021-gene panel sequencing. Non-synonymous SNVs and Indels with ≥3% allele frequency were included in the calculation of TMB. TMB-high (TMB-H) was defined as ≥9 Mutations/MB (Muts/MB) (16).

mTBI (molecular tumor burden index): Select the mutations of a specific sample with VAF and more than 70% of matched MVAF as clonal mutations, then calculate the average VAF of those mutations as the mTBI of this sample.

The mTBI decrease rate was defined as (mTBI of pre-treatment plasma – mTBI of post-treatment plasma)/mTBI of pre-treatment, which was used to describe the degree to which the mutations of a patient were cleared after treatment.

### Statistical Analysis

The Mann–Whitney test and Wilcoxon test were used to estimate differences between two groups. The linear association between two continuous data groups was assessed using Pearson correlation. The statistics were performed using GraphPad Prism 7 (GraphPad Software, La Jolla, CA, USA). Results were considered statistically significant when *p*-value < 0.05.

## Results

### Patient Characteristics

Tumor tissue samples, BWF (separated as supernatant and precipitate), and pre-treatment plasma samples were collected from 21 ES-SCLC patients enrolled in this study before receiving chemotherapy, and post-treatment plasma samples were collected from 14 patients. All the samples were subjected to next-generation sequencing (NGS) using a 1021-gene panel. For one patient, the extraction of DNA from BWFs was failed, and for another patient the extraction of DNA from pre-treatment plasma samples and post-treatment plasma was failed. Twenty BWFs samples, 21 BWFp samples, 21 tumor tissue samples, 20 pre-treatment plasma samples, and 13 post-treatment plasma samples were successfully tested. Supplementary Figure 1 shows the overall study design. Table 1 shows the patient characteristics of the 21 patients in this study. For these patients, the median age was 64 years, and the majority (71.4%) were male. Overall, 8 of 21 (38.1%) patients had stage IV disease, 5 of 21 (23.8%) had family history, and 15 of 21 (71.4%) had smoking history. The incidence rate of hyponatremia in these patients was 19.0% (4/19).

**Table 1:** Patient characteristics.

**Characteristic**	**Number**	**Percentage (%)**
**Age (years)**		
Median	64 (46, 77)	
**Sex**		
Male	15	71.4
Female	6	28.6
**Clinical stage**		
III	13	61.9
IV	8	38.1
**Family history**	5	23.8
**Smoking history**		
Current/former	15	71.4
Never	6	28.6
**Hyponatremia**		
Yes	4	19.0
No	17	81.0
**Tumor size**		
≤ 3 cm	1	4.8
>3 cm, ≤ 5 cm	5	23.8
>5 cm, ≤ 7 cm	9	42.8
>7 cm	6	28.6
**Tumor markers**		
ProGRP positive	18	85.7%
NSE positive	11	52.4%
CEA positive	12	57.1%

### Mutation Characteristics

Five types of matched samples from 21 patients were analyzed by NGS. The cfDNA concentration of BWFs ranged from 3.5 to 612.0 ng/ml (mean, 125.4 ng/ml), while the plasma cfDNA concentration in our study ranged from 2.4 to 768.4 ng/ml (mean, 99.6 ng/ml); the average plasma cfDNA concentration in healthy people was 5.77 ng/ml according to the GENE database. Compared with plasma cfDNA from healthy and SCLC patients, the concentration of BWFs cfDNA from SCLC patient was higher (one case failed).

**Figure 1:**
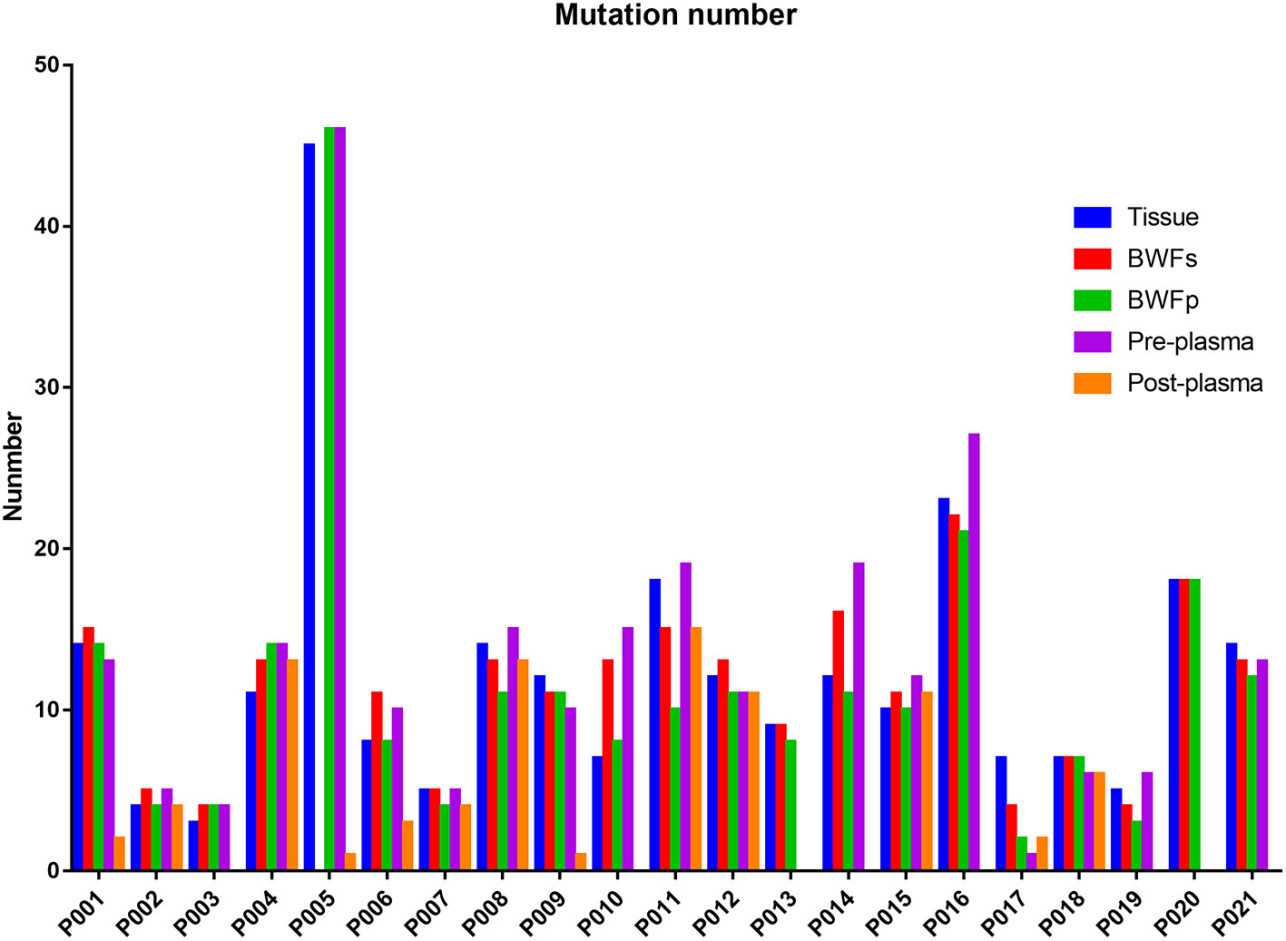
Mutation number in different sample types for each patient.

**Figure 2:**
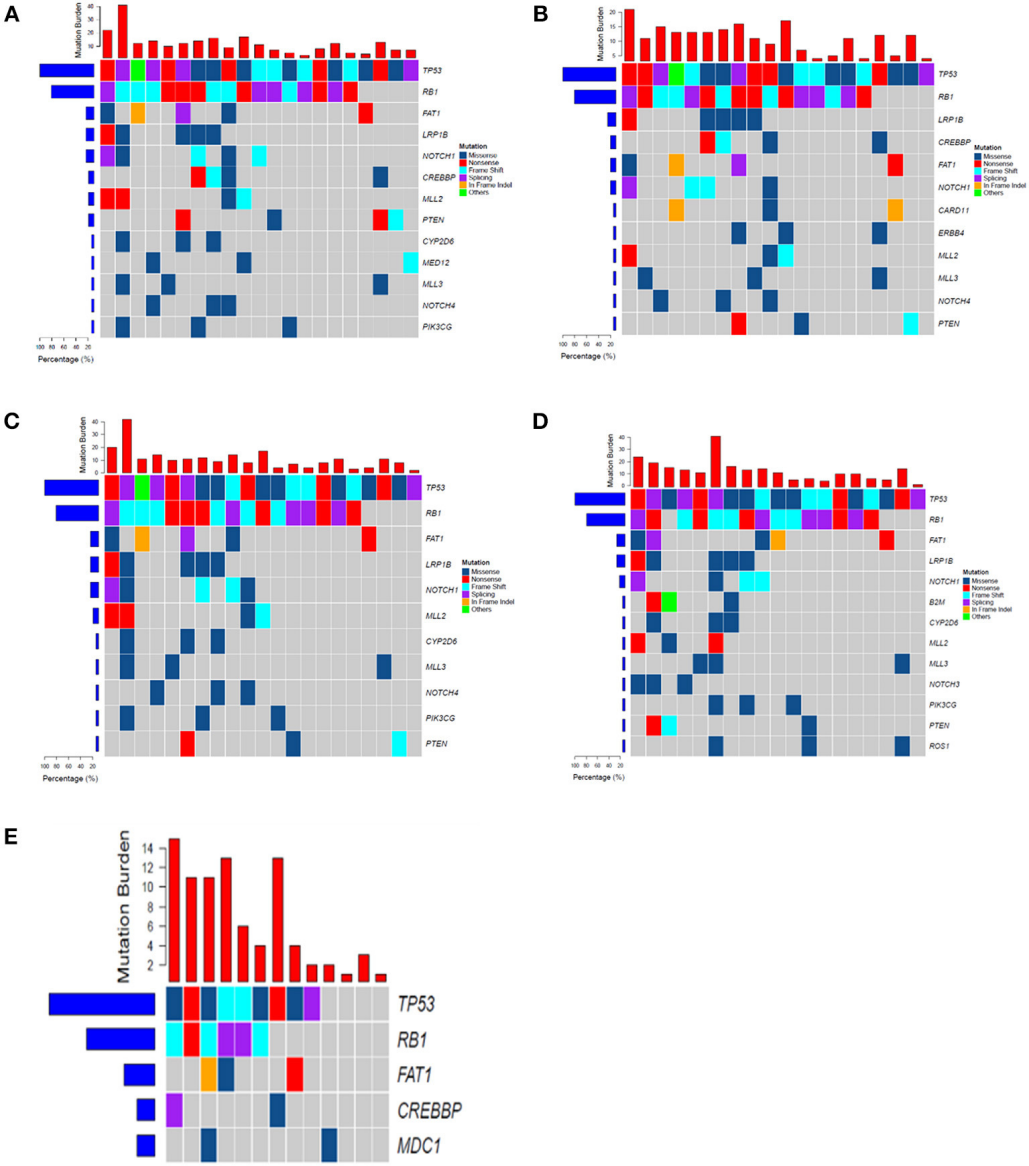
The frequency spectrum for all patients with detectable mutations. **(A)** Mutation frequency spectrum in tumor tissue (only showed genes existing in more than three samples). **(B)** Mutation frequency spectrum in BWFs (only showed genes existing in more than three samples). **(C)** Mutation frequency spectrum in BWFp (only showed genes existing in more than three samples). **(D)** Mutation frequency spectrum in pre-treatment plasma (one sample was not detected; only showed genes existing in more than three samples). **(E)** Mutation frequency spectrum in post-treatment plasma (only showed genes existing in more than two samples).

**Figure 3:**
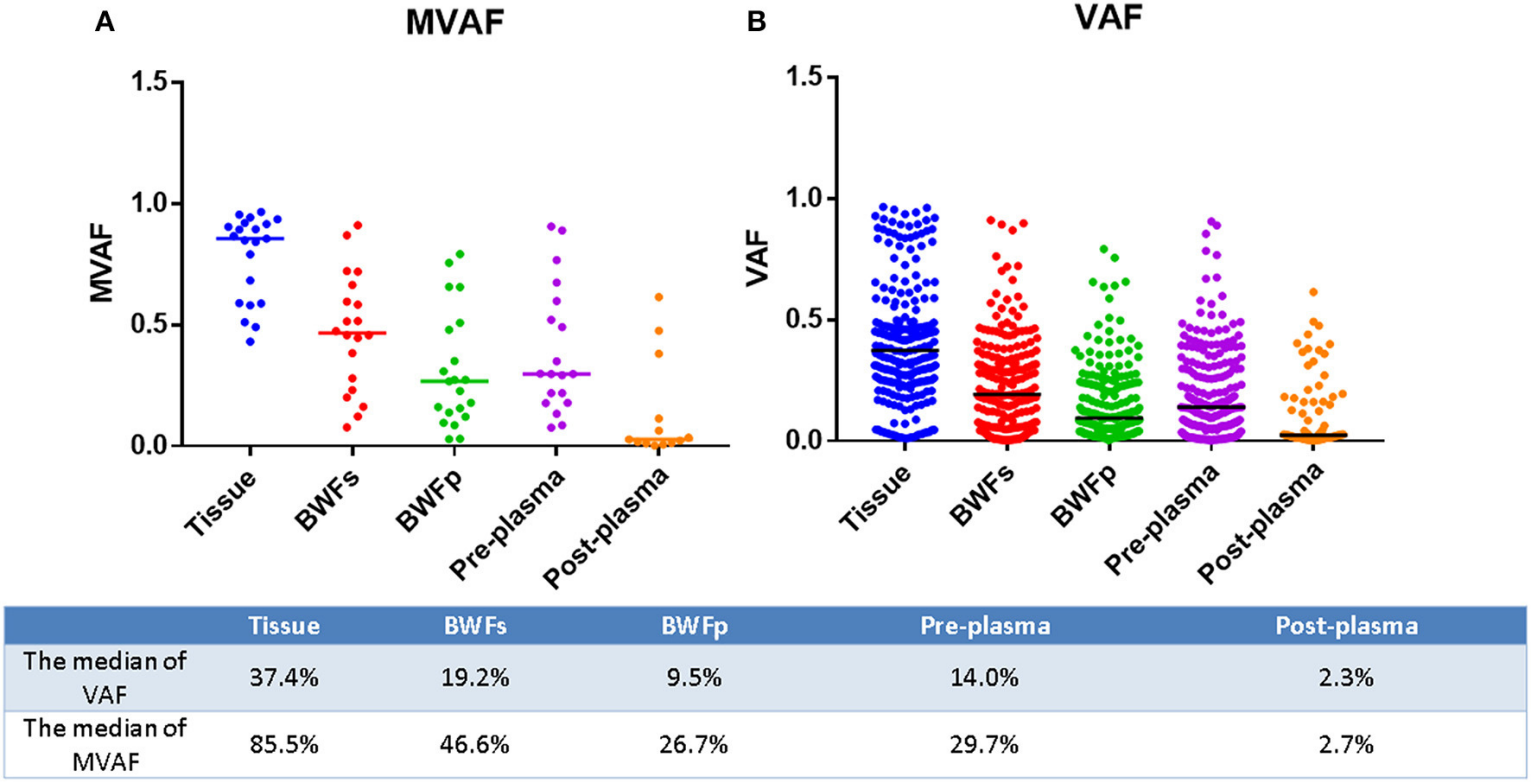
MVAF and VAF in different sample types for each patient. **(A)** MVAFs of five types of samples. **(B)** VAFs of five types of samples.

Mutations were identified in 20 (100%) of BWFs samples and 21 (100%) of BWFp samples, 21 (100%) of tumor tissues, 19 (95%) of pre-treatment plasma samples, and 13 (100%) of post-treatment plasma samples. Figure 1 shows the mutation number in different sample types for each patient. In tumor tissue samples, the most recurrent mutations were observed in the *TP53* gene, followed by *RB1*, which is the same as other sample types (Figure 2). The VAFs of tissue samples were significantly higher than those of the other three types of pre-treatment sample (paired *t*-test, all *p* < 0.0001). For biofluid samples, compared with pre-treatment plasma samples, the VAF of BWFs samples was significantly higher (paired *t*-test, *p* = 0.003). BWFp samples had relatively lower VAF than the other three types of pre-treatment sample (paired *t*-test, all *p* < 0.0001). As for MVAF, the result was similar, except that the VAFs of BWFp and pre-treatment plasma had no significant difference (Figure 3). In addition, mutations were detected in tissue samples and BWFs samples of three TBNA patients, including recurrent genes like *TP53* and *RB1*. For pre-treatment plasma samples, mutations were detected in only one patient, and one in three TBNA patients had no pre-treatment plasma sample.

The number of mutations in the post-treatment plasma was significantly less than that in the pre-treatment plasma (Wilcoxon matched-pair signed-rank test, *p* = 0.0059, Figure 1). Then, we compared VAF and MVAF between post-treatment plasma and pre-treatment plasma. The results showed that VAF and MVAF in post-treatment plasma were less than those in pre-treatment plasma (Wilcoxon matched-pair signed-rank test, *p* < 0.0001, *p* = 0.0012, respectively, Figure 3).

Then, we calculated the mTBI of BWFs and plasma samples and evaluated the mTBI changes after treatment. First, we tried to find if the mTBI of BWFs and pre-treatment plasma could predict the prognosis of patients. Therefore, we applied a survival analysis based on mTBI of BWFs and pre-treatment plasma. The results showed that the patients with lower mTBI of BWFs had a longer OS than those with higher mTBI of BWFs (log-rank test, *p* = 0.048). As for progression-free survival (PFS) and other factors, there were no statistically significant results.

As for the ctDNA dynamics, the results showed that the mTBI of plasma of each patient significantly decreased after treatment (Wilcoxon matched-pair signed-rank test, *p* = 0.0017). Meanwhile, we calculated the mTBI decrease rate of each patient and explored if it was associated with the prognosis of patients. However, there were no statistically significant results found (log-rank test, *p* > 0.05).

Among those 21 patients, 17 got a partial response (PR) status after treatment and four got a progressive disease (PD) status. All of the patients who had post-treatment plasma and were tested got PR, which was in accordance with the variation tendency of mutations in plasma.

### Concordance of Different Types of Pre-treatment Samples

**Figure 4:**
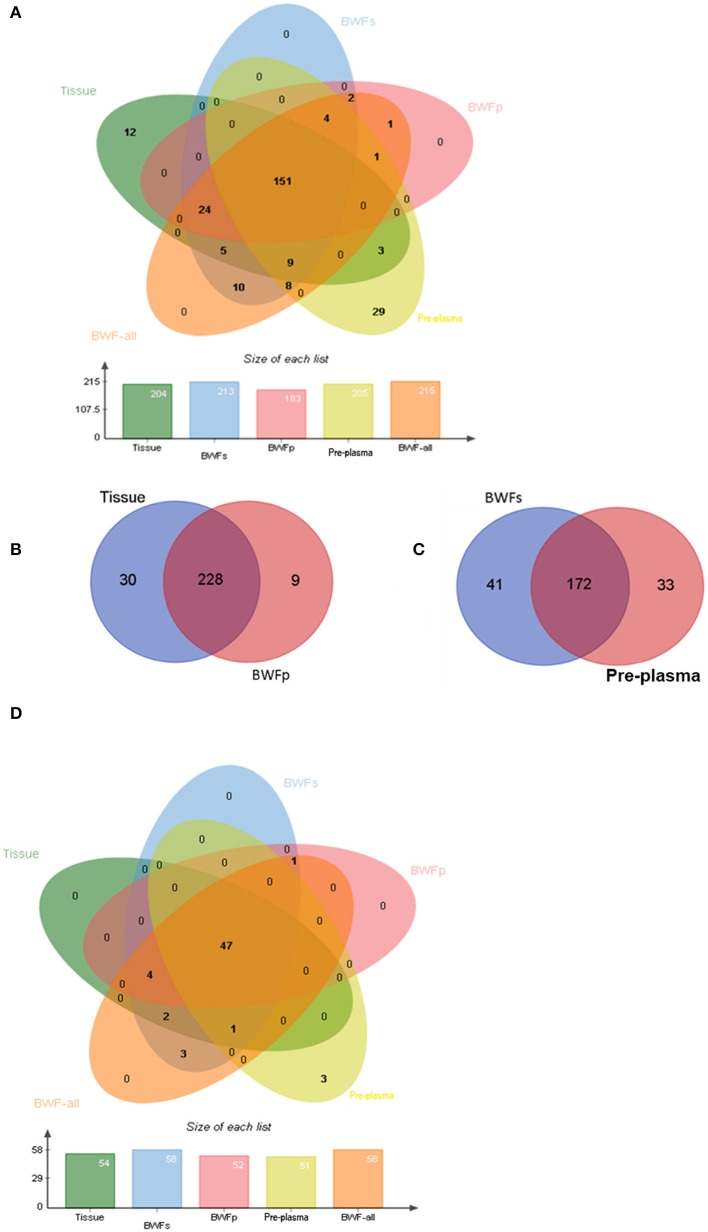
Genomic concordance of different matched types of samples. **(A)** Mutations detected in four types of sample. **(B)** Mutations detected in tissues and BWFp samples. **(C)** Mutations detected in BWFs samples and plasma samples. **(D)** The concordance between driver gene mutations detected in four types of sample.

Nineteen of the 21 tissue samples had matched BWFs, BWFp, and pre-treatment plasma samples. The concordance analysis was conducted with these 19 matched pre-treatment samples. For these samples, 204 genomic alterations were identified in tissue samples. In the meanwhile, 189 (92.6%), 175 (85.5%), and 163 (79.9%) alterations were detected in the matched BWFs, BWFp, and pre-treatment plasma samples, respectively. For the 19 patients, there were 213 alterations detected in BWF (BWFs and BWFp) samples, which was more than that in tissue samples. The BWF shows potential capability of detecting mutations (Figure 4A).

In the 21 tissue and BWFp-paired samples, 267 alterations were detected, of which 258 were detected in tissue samples and 237 in BWFp. The same alterations were 85.4% (228/267). Nine alterations in BWFp were not detected in tissues, and 30 mutations in tissues were not in BWFp (Figure 4B). In the 21 pre-treatment plasma samples and BWFs-paired samples, 246 alterations were detected, of which 205 were detected in pre-treatment plasma and 213 in BWFs. The same alterations were 69.9%. Forty-one alterations were not detected in BWFs, and 33 alterations were not in pre-treatment plasma (Figure 4C).

For driver mutations, the concordance between mutations in tissue and BWF (BWFs and BWFp) samples were higher than the concordance between tissue and pre-treatment plasma samples. The concordance of BWFs, BWFp, and pre-treatment plasma were 100% (54/54), 94.4% (51/54), and 88.9% (48/54), respectively (Figure 4D). This result showed the advantage of BWF in detecting driver mutations, which demonstrated the value of BWF in clinical application.

### Comparison Between Biochemical Tumor Markers and Genetic Tumor Markers

Mutations were identified in 20 (100%) of BWFs samples, 21 (100%) of BWFp samples, 21 (100%) of tumor tissues, and 19 (95%) of pre-treatment plasma. It indicates that sequencing has a high sensitivity. In contrast, we found that the sensitivity of three types of biochemical tumor markers associated with SCLC [progastrin-releasing peptide (ProGRP), neuron-specific enolase (NSE), and carcinoembryonic antigen (CEA)] tested before treatment was relatively lower. The percentages of patients that were tested as positive by tumor markers were 85.7% (ProGRP, 18/21), 52.4% (NSE, 11/21) and 57.1% (CEA, 12/21), respectively, which demonstrated that the sequencing results of four types of pre-treatment samples were more reliable for cancer detection.

### Tumor Mutational Burden

Moreover, tumor mutational burden (TMB), which was a molecular biomarker that can be used in immunotherapy efficacy prediction, was calculated. Table 2 shows the TMB status in the different types of pre-treatment samples. According to quartiles of TMB in our database, we define TMB-H as more than or equal to nine mutations per megabase. The proportion of TMB-H samples was separately 61.9% (13/21) in tissue samples, 60% (12/20) in BWFs samples, and 52.38% (11/21) in BWFp samples.

**Table 2:** The TMB status in the different types of samples.

**TMB (Muts/Mb)**	**Number (tissue)**	**Number (BWFs)**	**Number (BWFp)**	**Number (pre-plasma)**
≤ 6	6/21	6/20	7/21	7/20
>6, ≤ 9	3/21	2/20	4/21	0/20
>9, ≤ 20	10/21	11/20	9/21	11/20
>20	2/21	1/20	1/21	2/20

We measured the tumor mutation burden (TMB) by our comprehensive genomic panel (targeting 1,021 genes, 1.6 Mb of genome). We conducted the targeting sequencing on the four sample types of the same patients, then analyzed and calculated the number of somatic non-synonymous mutations per megabase of genomic coverage.

**Figure 5:**
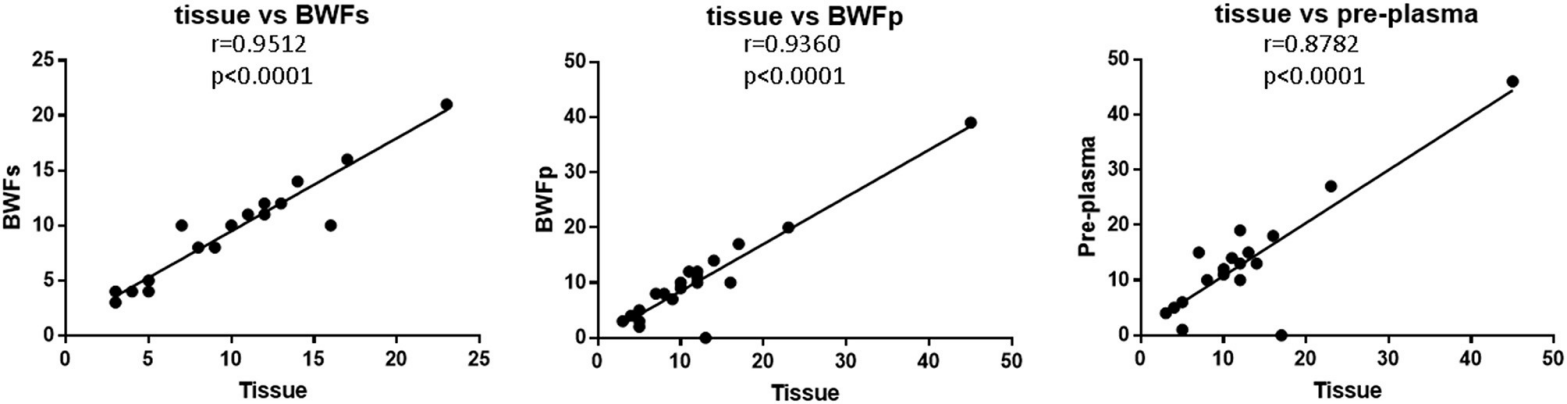
Correlation of TMB in different types of pre-treatment sample.

The TMB of BWFs, BWFp, and pre-treatment plasma samples all had strong correlation with that of tissue samples (Figure 5). The correlation between BWFs TMB and tissue TMB was the strongest (Pearson *r* = 0.9512, *p* < 0.0001), followed by the correlation of BWFp TMB and tissue TMB (Pearson *r* = 0.9360, *p* < 0.0001). The correlation between pre-treatment plasma TMB and tissue TMB was the lowest (Pearson *r* = 0.8782, *p* < 0.0001) but still shows a strong correlation.

### Relationship Between Clinical Characteristics and Mutations

There were four patients diagnosed as hyponatremia (low sodium, LoNa) in those 21 patients. For both hyponatremia patients and others, the most frequently mutated genes were *RB1* and *TP53* which were typically mutated genes in SCLC. No unique mutation was found in four patients with hyponatremia relatively to non-hyponatremia patients.

**Figure 6:**
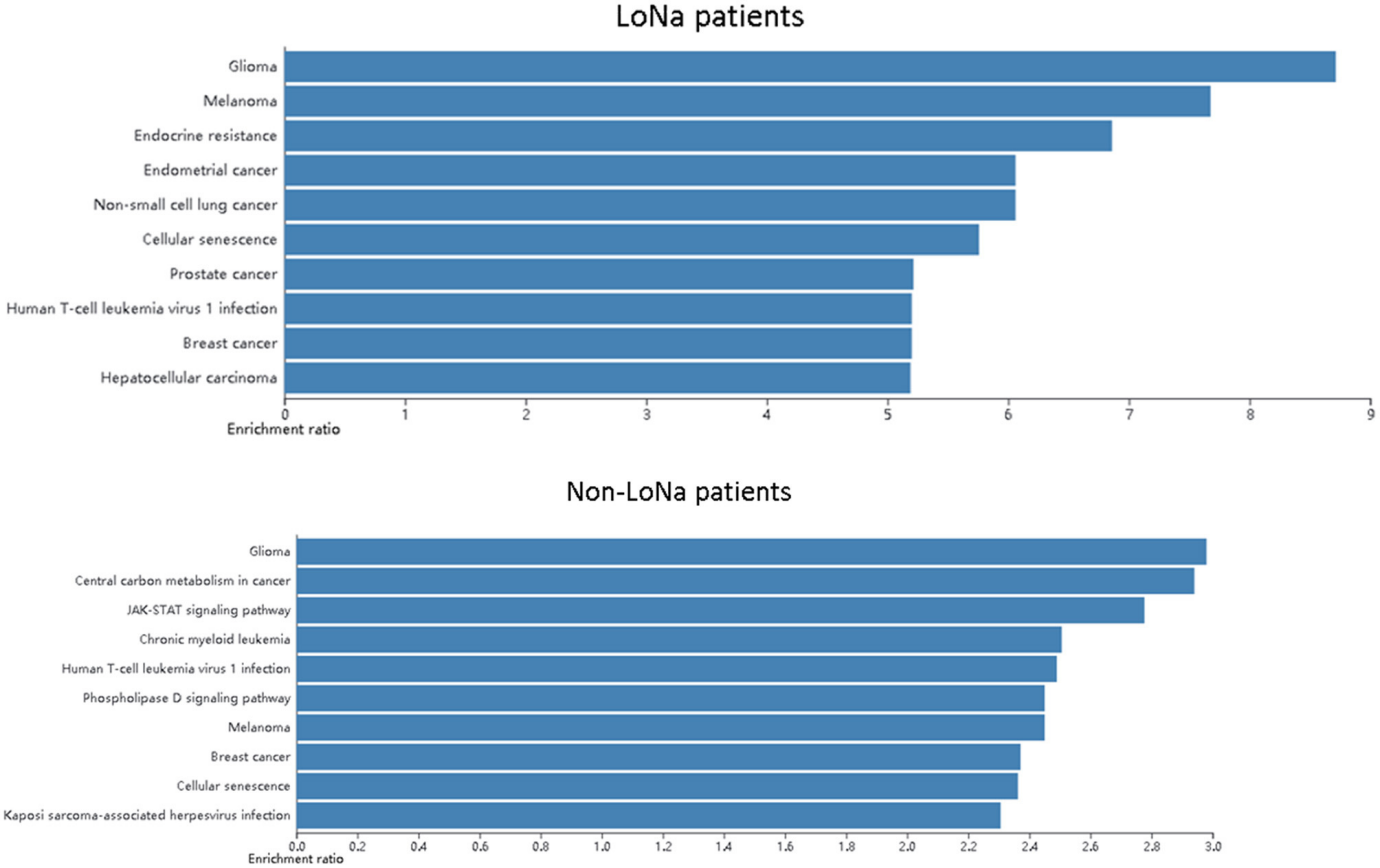
Pathway analysis of LoNa patients and non-LoNa patients.

Then a pathway analysis was applied to find if LoNa was related to specific alterations of the molecular pathway. It is notable that the endocrine resistance pathway was found enriched in hyponatremia patients which may be related to hyponatremia (Figure 6).

We also analyzed the correlation between tumor size and MVAF. However, there was no statistically significant correlation between them (Pearson correlation, *p* > 0.05).

In addition, we tried to explore the relationship between mutated genes and OS/PFS. Therefore, we performed the univariate/multivariate Cox regression analysis to find the genes which were relevant to the prognosis of patients to a certain extent (*p* ≤ 0.2. By univariate Cox regression, we found 12 genes which were related to patients' PFS and 24 genes related to patients' OS. However, when we apply those genes to multivariate Cox analysis, none of those genes were significant with all *p* ≥ 0.05.

## Discussion

With the rapid development of molecular testing and liquid biopsies, the therapy strategies for lung cancer become more individualized. Despite significant advances in clinical treatment of non-small cell lung cancer, SCLC has been recognized as a drug development blind spot. The key to accurate oncology is the identification and detection of molecular biomarkers that can predict the diagnosis, prognosis, and curative effect (17). Therefore, one of the priorities for future clinical research on SCLC is to seek predictive markers for accurate treatment. NGS, which provides a highly sensitive and accurate high-throughput platform for large-scale genome testing, as well as liquid biopsy, which greatly improves the convenience of gene detection by using ctDNA, has been widely used in cancer studies. The wide application of these techniques has provided a broad prospect for the further exploration of SCLC. Although surgical biopsy is still the “gold standard” for diagnosis and treatment (18), there are many limitations of tissue biopsy in clinical practice due to the location and heterogeneity of the tumor and the intolerance of the patient (19, 20). Liquid biopsy is a simple, easy-to-be-repeated, and less invasive method, and it has the potential benefits as a parallel approach, or even a standalone approach, for later lines of treatment (8, 10). In lung cancer, liquid biopsy using plasma ctDNA has been widely used in clinical practice, and its feasibility has been shown in many studies. In addition to plasma being used for liquid biopsy, other body fluids such as urine, pleural fluid, cerebrospinal fluid, and bronchial washing fluid can also be used for exploration (10).

BW is a technique developed on fiber-optic bronchoscopy, which can directly obtain the exfoliated specimens and secretion of the distal pathological tissues, and BWF may contain DNA shed from tumor cells (21). Considering the direct adjacency to bronchi, BWF might be a potential alternative to biopsy specimens. BWF close to a tumor or metastasis may contain more abundant and less diluted tumor DNA with higher concentrations, compared to what can be isolated from the plasma (10). Many studies have explored the feasibility of molecular detection using BWF for the diagnosis and predictive assessment of lung cancer (22–24). One study has shown that compared with liquid biopsy using plasma, using BWF resulted in higher diagnostic yields for detecting mutations in lung cancer (23). However, there is still lack of studies showing the landscape of mutations in BWF and their concordance with mutations in tissues or plasma in ES-SCLC. In this study, we evaluated the potential use of BWF in liquid biopsy in ES-SCLC. BWF (BWFs and BWFp), tumor tissues, pre-treatment plasma samples, and post-treatment plasma samples were detected by NGS in 21 ES-SCLC patients. The consistency of the alterations between BWF and tissue was higher than that between pre-treatment plasma and tissue, and that of BWFs was higher than that of BWFp. Mutations not detected in tissue or pre-treatment plasma were detected in the BWF, which suggested that the detection of BWF could supplement the detection of tissue and plasma and make the obtained mutation data more comprehensive. For patients who had no primary lesions under bronchoscopy, the mutation can still be detected with BWF. Alteration consistency analysis indicates that the BWF can be used as a supplement for tissue and plasma. For advanced patients with inaccessible tissue biopsy, BWF might provide an alternative way in identifying DNA alterations. Moreover, BWFs samples have a much higher positive detection rate and higher VAF than BWFp samples. Driver mutations were detected, by NGS, in 100 and 94.4% of BWFs and BWFp, respectively. The abundance of tumor cells is critical for mutation detection in BWFp, but BWFp which was composed of immune cells and a few tumor cells may have limited tumor cell content. In contrast, BWFs was less affected by the presence of non-tumor cell components and may contain a more abundant and less analytically demanding tumor DNA (10). Thus, BWFs has a higher mutation detection sensitivity than BWFp and therefore is a more reliable source for genetic testing. A previous study using ctDNA to analyze the genomic evolution of the SCLC showed that ctDNA dynamic monitoring is highly compatible with imaging monitoring as a means of dynamic detection in patients with SCLC (12). Similar results were obtained in our study, which showed that VAF and MVAF of mutations in post-treatment plasma were less than those in pre-treatment plasma of patients with radiographic remission. It indicates that ctDNA dynamic change is consistent with radiographic-based outcome assessments. To explore the ctDNA dynamic change, we used an indicator mTBI for evaluation. We calculated the mTBI of BWFs and plasma samples and evaluated the mTBI changes after treatment. The mTBI of plasma of each patient significantly decreased after treatment, but the mTBI of the plasma decrease rate of each patient was not associated with the prognosis of patients. In our study, it was proved that the genomic alterations of BWF are highly consistent with those of plasma. BWF is easier to be repeated than tissue and contains more abundant tumor DNA than plasma, so it might be used for dynamic monitoring in the future. Therefore, we may be able to apply the sequencing of BWF for curative effect monitoring in the future. The monitoring parameter of BWF and ctDNA will further verify and standardize. Moreover, the patients with lower mTBI of BWFs had a longer OS than those with higher mTBI of BWFs. This indicates that mTBI of BWFs may be used as an indicator to judge the prognosis for SCLC patients. We will include more patients to confirm this conclusion in the future.

ProGRP, NSE, and CEA are important conventional tumor markers of SCLC. They are often used in the differential diagnosis of SCLC and the monitoring of late SCLC chemotherapy. However, the specificity of ProGRP, NSE, and CEA in the diagnosis and monitoring of SCLC is not high. We found that the sensitivity of three tumor markers before treatment was lower than that of sequencing, including sequencing with BWF. Thus, it may be possible to use the sequencing with BWF for SCLC screening and adjuvant diagnosis in the future, but the criteria and the specificity still need to be further explored.

Hyponatremia is one of the most common electrolyte disorders in cancer patients, whose main mechanism is abnormal secretion of anti-diuretic hormone (SIADH) caused by the increasing anti-diuretic hormone because of tumor cell secretion or application of related drugs (25, 26). SIADH is found in 1–2% of patients with malignant tumors, 10% of whom are SCLC (27). Hyponatremia is the main clinical feature of SIADH. The pathway analysis showed that the endocrine resistance pathway was found enriched in hyponatremia patients. So, hyponatremia is probably related to specific alterations of the molecular pathway. It is notable that this may reveal the molecular mechanism of hyponatremia.

Many new SCLC-treatment approaches have been explored, but none of the chemotherapy regimens has been superior to the standard EP regimen. In the Impower133 and Caspian study, chemotherapy in combination with immunotherapy as a first-line treatment achieved a breakthrough. However, the curative effect was heterogeneity and it is urgent to recognize its curative benefit to people (3, 4). An accurate biomarker has become a major focus of current research. In non-small cell lung cancer (NSCLC), clinical efficacy is associated with the expression of PD-L1 on tumor cells (TCs) and tumor-infiltrating immune cells (ICs) (27). In NSCLC patients, the objective remission rate (ORR) was 23% in patients with TCS and/or ICs with a high PD-L1 expression (28). However, the frequency of PD-L1 expression in SCLC is reported to be lower than in NSCLC (29). A retrospective analysis of biomarkers from the CheckMate 032 study showed that SCLC patients with high TMB had better improvement in ORR, OS, and PFS when treated with Nivolumab alone or Nivolumab in combination with Ipilimumab than patients with low TMB (30). SCLC is known to have a relatively high TMB [median ~8 mutations per megabase (mut/Mb)] (31). In our study, compared with the proportion of TMB-H samples in tissue samples counting 61.9% (13/21), 60% (12/20) of BWFs samples and 52.38% (11/21) of BWFp samples were TMB-H (defined as more than or equal to nine mutations per megabase). The TMBs of BWFs, BWFp, and pre-treatment plasma samples all had a strong correlation with those of tissue samples. The TMB of BWFs had the strongest correlation, and the TMB of pre-treatment plasma had a relatively lower correlation compared with those of BWFs and BWFp. In our study, TMB of BWF had a strong correlation with that of tissue samples. Thus, it is possible to find a new way to detect biomarkers for immunotherapy. Next, we will further explore the correlation between TMB grade in BWF and first-line SCLC immunotherapy.

Our study showed that BWF, especially BWFs, can capture the genetic information of ES-SCLC patients as a complement for tumor tissue and peripheral blood biopsies. Genetic markers such as prognostic and ICB-related marker from BWF are potential clinical practice markers. However, further studies with larger sample sizes are needed.

## Conclusions

For ES-SCLC patients, the liquid biopsy of BWF showed high potential in identifying DNA alterations and calculating TMB, which suggested that genomic analysis of BWF liquid biopsy may have clinical value in seeking predictive markers. It can be widely used in routine clinical practice, which can be easily repeated. With the development of specimen collection technology and high-sensitivity detection technology, the biological information of SCLC can be comprehensively interpreted, and the mechanism of mutational genes can be deeply studied, which is beneficial to the research and development of accurate treatment for SCLC.

## Data Availability Statement

The data presented in the study are deposited in the European Variation Archive (EVA) repository. Project: PRJEB4372. Analyses: ERZ1758452.

## Ethics Statement

The studies involving human participants were reviewed and approved by the institutional review board of Shanxi Provincial Cancer Hospital. The patients/participants provided their written informed consent to participate in this study.

## Author Contributions

All authors listed have made a substantial, direct and intellectual contribution to the work, and approved it for publication.

## Conflict of Interest

ZL and YX were employed by company Geneplus-Beijing. The remaining authors declare that the research was conducted in the absence of any commercial or financial relationships that could be construed as a potential conflict of interest.

## References

[1] Gazdar A Bunn P Minna J. Small-cell lung cancer: what we know, what we need to know and the path forward. Nat Rev Cancer. (2017) 17:725–37. 10.1038/nrc.2017.87 29077690

[2] Siegel RL Miller KD Jema A. Cancer statistics, 2016. CA Cancer J Clin. (2016) 66:7–30. 10.3322/caac.21332 26742998

[3] Paz-Ares L Dvorkin M Chen Y Reinmuth N Hotta K Trukhin D. Durvalumab plus platinum–etoposide versus platinum–etoposide in first-line treatment of extensive-stage small-cell lung cancer (CASPIAN): a randomised, controlled, open-label, phase 3 trial. Lancet. (2019) 394:1929–39. 10.1016/S0140-6736(19)32222-6 31590988

[4] Nishio M Sugawara S Atagi S Akamatsu H Sakai H Okamoto I. Subgroup analysis of Japanese patients in a phase III study of atezolizumab in extensive-stage small-cell lung cancer (IMpower133). Clin Lung Cancer. (2019) 20:469–76.e461. 10.1016/j.cllc.2019.07.005 31466854

[5] Hellmann MD Ciuleanu T-E Pluzanski A Lee JS Otterson GA Audigier-Valette C. Nivolumab plus ipilimumab in lung cancer with a high tumor mutational burden. New Engl J Med. (2018) 378:2093–104. 10.1056/NEJMoa1801946 29658845 PMC7193684

[6] Overman MJ Lonardi S Wong KYM Lenz HJ Gelsomino F Aglietta M. Durable clinical benefit with nivolumab plus ipilimumab in DNA mismatch repair-deficient/microsatellite instability-high metastatic colorectal cancer. J Clin Oncol. (2018) 36:773. 10.1200/JCO.2017.76.9901 29355075

[7] Wang Z Duan J Cai S Han M Dong H Zhao J. Assessment of blood tumor mutational burden as a potential biomarker for immunotherapy in patients with non–small cell lung cancer with use of a next-generation sequencing cancer gene panel. AMA Oncol. (2019) 5:696–702. 10.1001/jamaoncol.2018.7098 30816954 PMC6512308

[8] Corcoran R. Liquid biopsy versus tumor biopsy for clinical-trial recruitment. Nat Med. (2020) 26:1815–6. 10.1038/s41591-020-01169-6 33230339

[9] Rolfo C Cardona A Cristofanilli M Paz-Ares L Diaz Mochon J Duran I. Challenges and opportunities of cfDNA analysis implementation in clinical practice: perspective of the International Society of Liquid Biopsy (ISLB). Crit Rev Oncol Hematol. (2020) 151:102978. 10.1016/j.critrevonc.2020.102978 32428812

[10] Durin L Pradines A Basset C Ulrich B Keller L Dongay V. Liquid biopsy of non-plasma body fluids in non-small cell lung cancer: look closer to the tumor! Cells. (2020) 9:2486. 10.3390/cells9112486 33207539 PMC7698102

[11] Blackhall F Frese KK Simpson K Kilgour E Brady G Dive C. Will liquid biopsies improve outcomes for patients with small-cell lung cancer? Lancet Oncol. (2018) 19:e470–81. 10.1016/S1470-2045(18)30455-8 30191851

[12] Nong J Gong Y Guan Y Yi X Yi Y Chang L. Circulating tumor DNA analysis depicts subclonal architecture and genomic evolution of small cell lung cancer. Nat Commun. (2018) 9:1–8. 10.1038/s41467-018-05327-w 30082701 PMC6079068

[13] Devarakonda S Sankararaman S Herzog BH Gold KA Waqar SN Ward JP. Circulating tumor DNA profiling in small-cell lung cancer identifies potentially targetable alterations. Clin Cancer Res. (2019) 25:6119–26. 10.1158/1078-0432.CCR-19-0879 31300452 PMC7373320

[14] Kandoth C McLellan MD Vandin F Ye K Niu B Lu C. Mutational landscape and significance across 12 major cancer types. Nature. (2013) 502:333–9. 10.1038/nature12634 24132290 PMC3927368

[15] Jing W Suhas V Zhiao S Michael G Bing Z. WebGestalt 2017: a more comprehensive, powerful, flexible and interactive gene set enrichment analysis toolkit. Nucleic Acids Res. (2017) 45:W130–7. 10.1093/nar/gkx356 28472511 PMC5570149

[16] Zhang Y Chang L Yang Y Fang W Guan Y Wu A. The correlations of tumor mutational burden among single-region tissue, multi-region tissues and blood in non-small cell lung cancer. J Immunother Cancer. (2019) 7:1–5. 10.1186/s40425-019-0581-5 30944026 PMC6448263

[17] Bailey AM Mao Y Zeng J Holla V Meric-Bernstam F. Implementation of biomarker-driven cancer therapy: existing tools and remaining gaps. Discov Med. (2014) 17:101–14. 10.1590/1414-431X20133278 24534473 PMC4160907

[18] Domínguez-Vigil IG Moreno-Martínez AK Wang JY Roehrl MH Barrera-Saldaña HA. The dawn of the liquid biopsy in the fight against cancer. Oncotarget. (2017) 9:2912–22. 10.18632/oncotarget.23131 29416824 PMC5788692

[19] Bedard PL Hansen AR Ratain MJ Siu LL. Tumor heterogeneity in the clinic. Nature. (2013) 501:355–64. 10.1038/nature12627 24048068 PMC5224525

[20] Sholl LM Aisner DL Allen TC Beasley MB Cagle PT Capelozzi VL. Liquid biopsy in lung cancer: a perspective from members of the pulmonary pathology society. Arch Pathol Lab Med. (2016) 140:825–9. 10.5858/arpa.2016-0163-SA 27195432

[21] Schmidt B Carstensen T Engel E Jandrig B Witt C Fleischhacker M. Detection of cell-free nucleic acids in bronchial lavage fluid supernatants from patients with lung cancer. Eur J Cancer. (2004) 40:452–60. 10.1016/j.ejca.2003.10.020 14746865

[22] Ryu J Lim J Lee M Lee S Kim H Kim M. Feasibility of bronchial washing fluid-based approach to early-stage lung cancer diagnosis. Oncologist. (2019) 24:e603–6. 10.1634/theoncologist.2019-0147 31036768 PMC6656449

[23] Lee S Kim E Kim T Chang Y. Compared to plasma, bronchial washing fluid shows higher diagnostic yields for detecting EGFR-TKI sensitizing mutations by ddPCR in lung cancer. Respir Res. (2020) 21:142. 10.1186/s12931-020-01408-x 32517757 PMC7281949

[24] Roncarati R Lupini L Miotto E Saccenti E Mascetti S Morandi L. Molecular testing on bronchial washings for the diagnosis and predictive assessment of lung cancer. Mol Oncol. (2020) 14:2163–75. 10.1002/1878-0261.12713 32441866 PMC7463327

[25] Ellison DH Berl T. The syndrome of inappropriate antidiuresis. New Eng J Med. (2007) 356:2064–72. 10.1056/NEJMcp066837 17507705

[26] Hermes A Waschki B Reck M. Hyponatremia as prognostic factor in small cell lung cancer–a retrospective single institution analysis. Respir Med. (2012) 106:900–4. 10.1016/j.rmed.2012.02.010 22405607

[27] Herbst RS Soria J-C Kowanetz M Fine GD Hamid O Gordon MS. Predictive correlates of response to the anti-PD-L1 antibody MPDL3280A in cancer patients. Nature. (2014) 515:563–7. 10.1038/nature14011 25428504 PMC4836193

[28] Leora H Gettinger SN Gordon MS Herbst RS Leena G Enriqueta F. Safety and clinical activity of atezolizumab monotherapy in metastatic non-small-cell lung cancer: final results from a phase I study. Eur J Cancer. (2018) 101:201–9. 10.1016/j.ejca.2018.06.031 30077125

[29] Gelsomino F Lamberti G Parisi C Casolari L Ardizzoni A. The evolving landscape of immunotherapy in small-cell lung cancer: a focus on predictive biomarkers. Cancer Treat Rev. (2019) 79:101887. 10.1016/j.ctrv.2019.08.003 31491661

[30] Janjigian YY Bendell J Calvo E Kim JW Ascierto PA Sharma P. CheckMate-032 study: efficacy and safety of nivolumab and nivolumab plus ipilimumab in patients with metastatic esophagogastric cancer. J Clin Oncol. (2018) 36:2836–44. 10.1200/JCO.2017.76.6212 30110194 PMC6161834

[31] Alexandrov LB Nik-Zainal S Vvedge DC Aparicio SAJR. Signatures of mutational processes in human cancer. Nature. (2013) 500:415–21.A413. 10.1038/nature12477 23945592 PMC3776390

