# Supplementary figures and images for: Identifying Genomic Alterations in Small Cell Lung Cancer Using the Liquid Biopsy of Bronchial Washing Fluid
Source: Front Oncol. 2021 Apr 26;11:647216. doi: 10.3389/fonc.2021.647216 (PMC8110515; doi:10.3389/fonc.2021.647216)

mTBI.decrease.rate    — >0.901422319(4)    — <0.901422319(8)

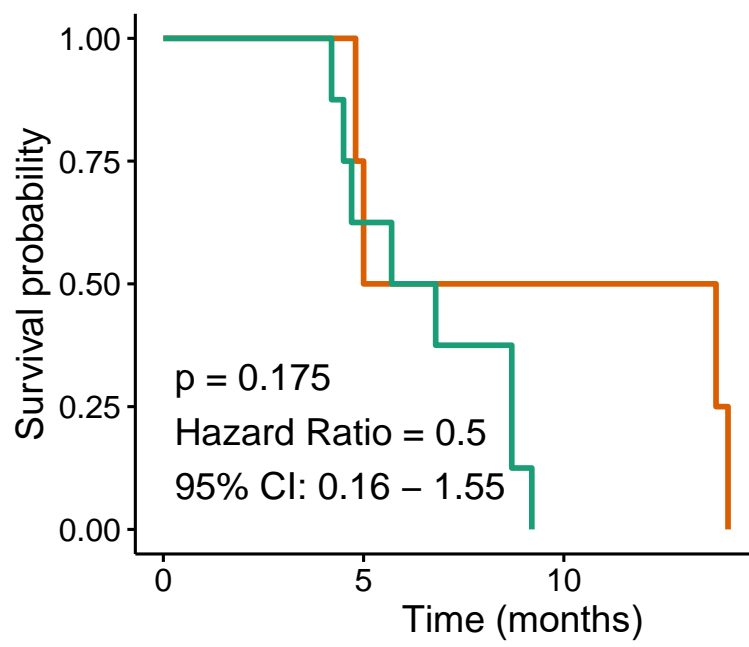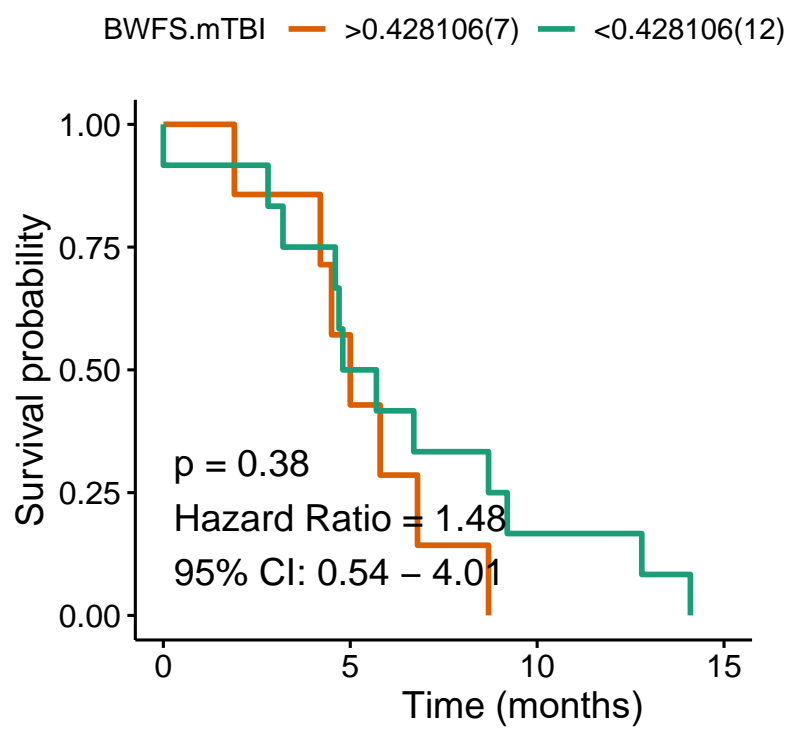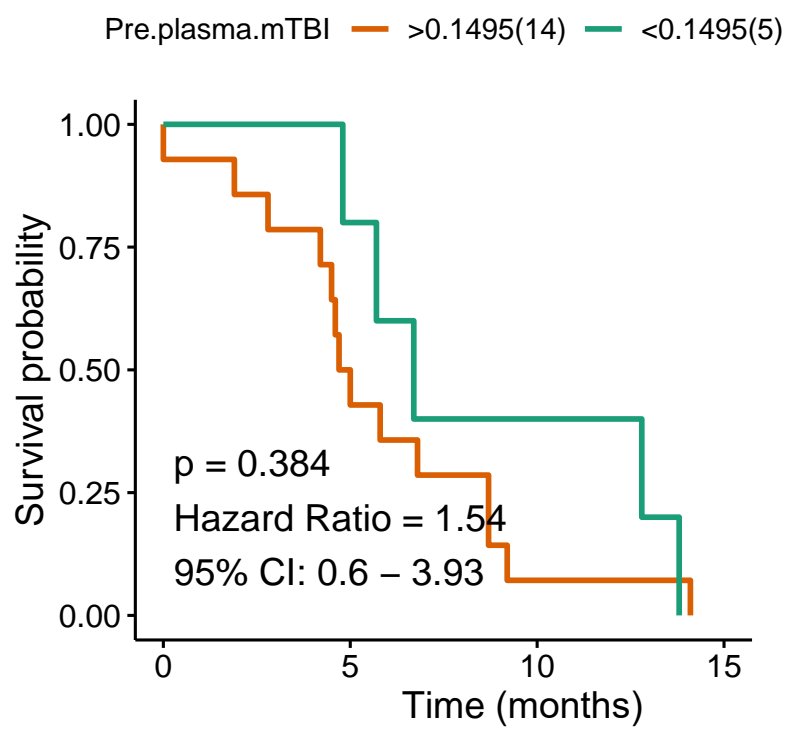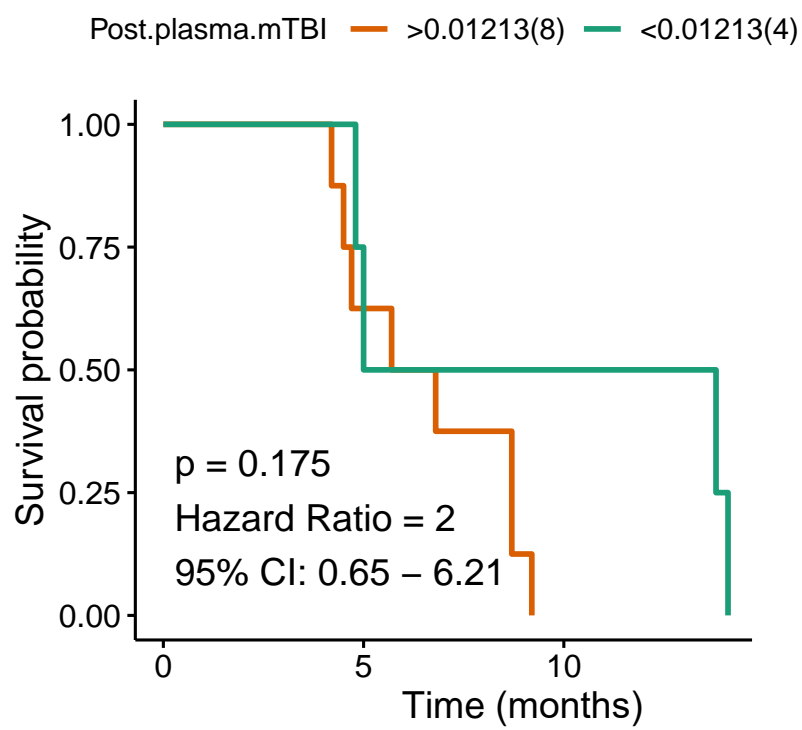

Supplement: Supplementary file 3 [file Data_Sheet_1.PDF]

mTBI.decrease.rate    — >0.901422319(4)    — <0.901422319(8)

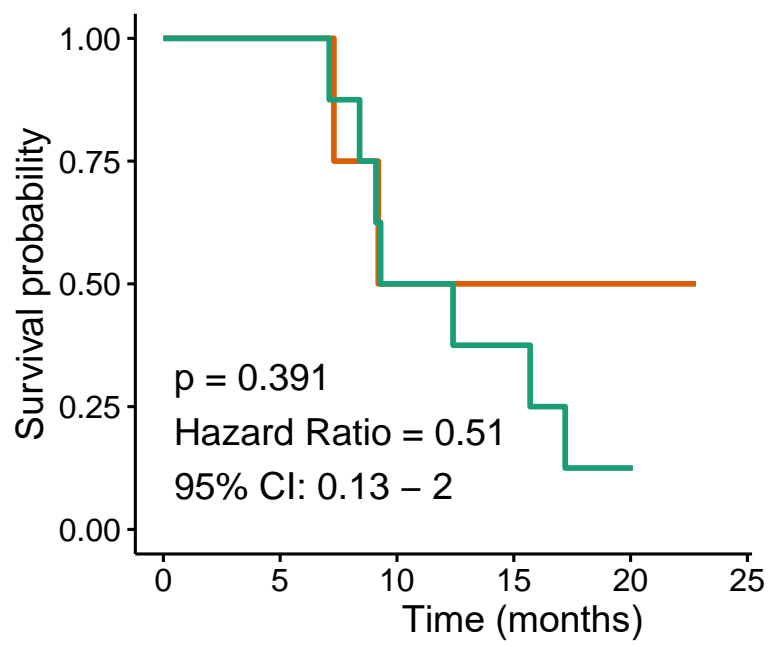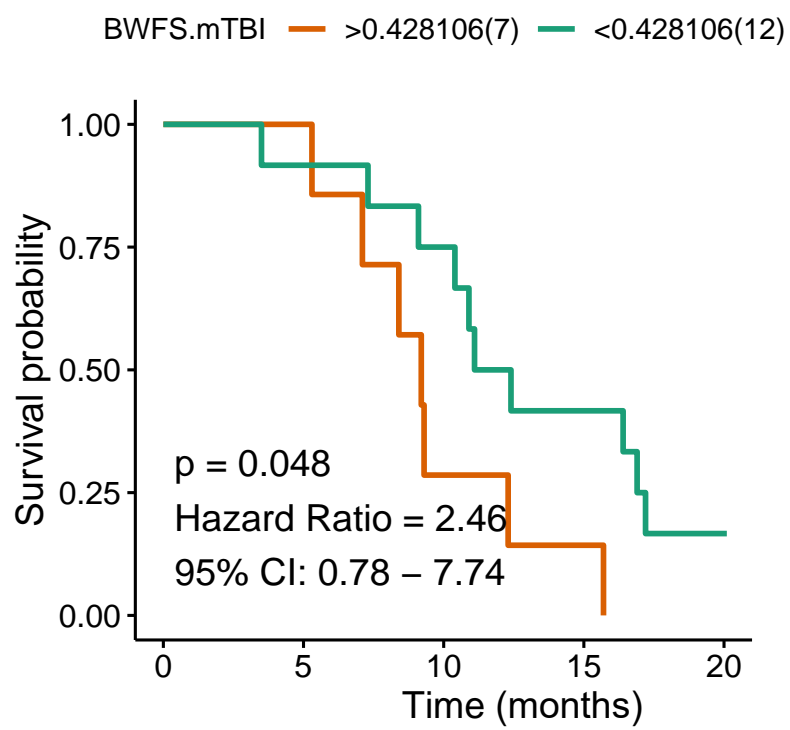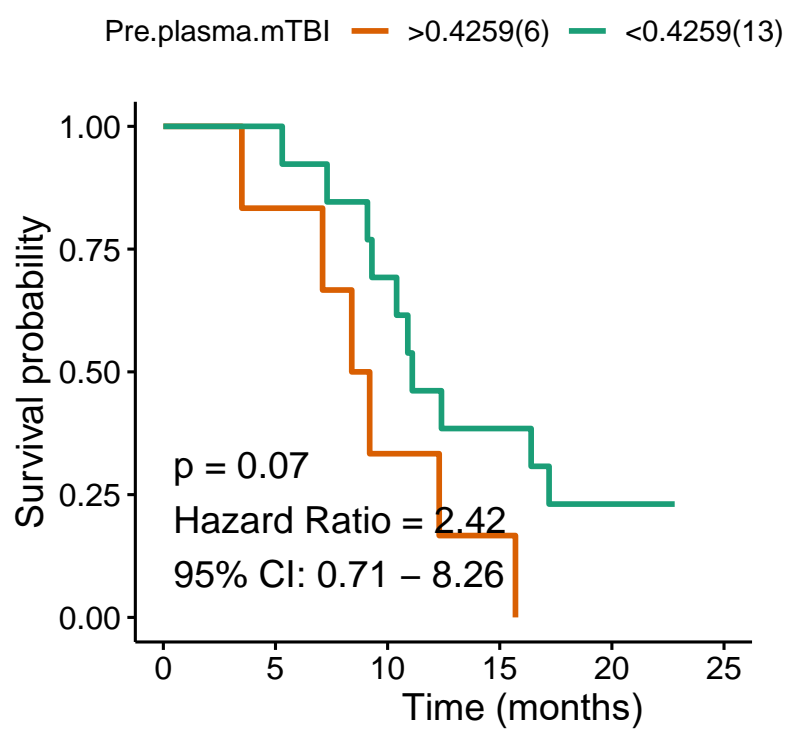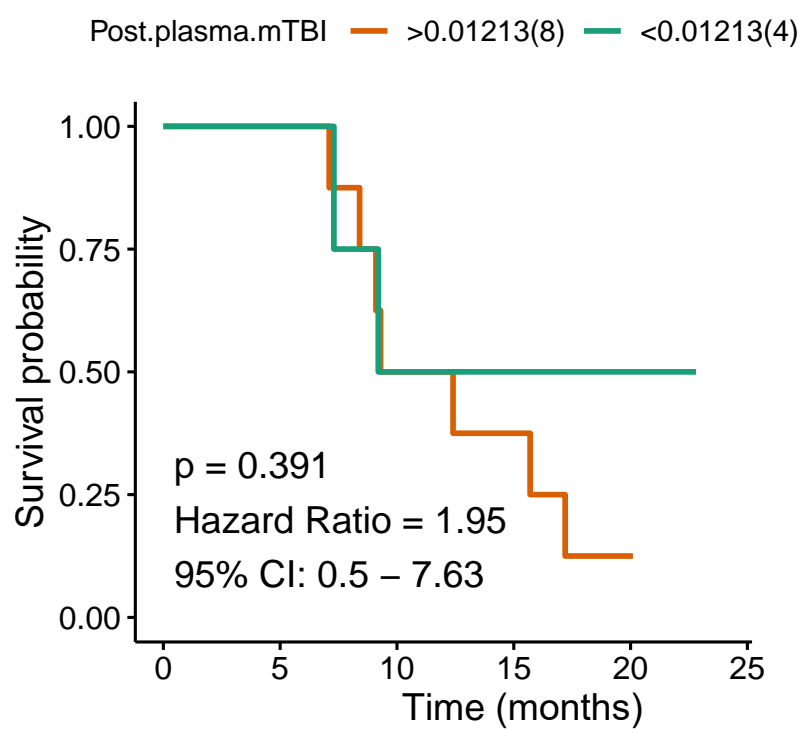

Supplement: Supplementary file 4 [file Data_Sheet_2.PDF]
